# Empathy Emerges Spontaneously in the Ultimatum Game: Small Groups and Networks

**DOI:** 10.1371/journal.pone.0043781

**Published:** 2012-09-26

**Authors:** Jaime Iranzo, Luis M. Floría, Yamir Moreno, Angel Sánchez

**Affiliations:** 1 Centro de Astrobiología (INTA- Consejo Superior de Investigaciones Científicas), Torrejón de Ardoz, Spain; 2 Instituto de Biocomputación y Fsica de Sistemas Complejos, Universidad de Zaragoza, Zaragoza, Spain; 3 Departamento de Fsica de la Materia Condensada, Universidad de Zaragoza, Zaragoza, Spain; 4 Departamento de Fsica Teórica, Universidad de Zaragoza, Zaragoza, Spain; 5 GISC, Matemáticas, Universidad Carlos III de Madrid, Leganés, Spain; University of Maribor, Slovenia

## Abstract

The Ultimatum game, in which one subject proposes how to share a pot and the other has veto power on the proposal, in which case both lose everything, is a paradigmatic scenario to probe the degree of cooperation and altruism in human subjects. It has been shown that if individuals are empathic, i.e., they play the game having in mind how their opponent will react by offering an amount that they themselves would accept, then non-rational large offers well above the smallest possible ones are evolutionarily selected. We here show that empathy itself may be selected and need not be exogenously imposed provided that interactions take place only with a fraction of the total population, and that the role of proposer or responder is randomly changed from round to round. These empathic agents, that displace agents with independent (uncorrelated) offers and proposals, behave far from what is expected rationally, offering and accepting sizable fractions of the amount to be shared. Specific values for the typical offer depend on the details of the interacion network and on the existence of hubs, but they are almost always significantly larger than zero, indicating that the mechanism at work here is quite general and could explain the emergence of empathy in very many different contexts.

## Introduction

Understanding cooperation is a major challenge in fields ranging from genetics and cell biology to evolutionary anthropology and behavioral economics [1]. Major advances on this issue date back to almost 50 years ago, and were based on genetic relatedness (kin selection [2]) and on the logic of repeated interactions (reciprocity [3]). These explanations of cooperative behavior have been extremely successful in different contexts and levels, including bacteria, social insects or humans, to name a few [4]–[6]. However, human cooperation is unique in so far as it appears in situations where those explanations are not plausible, such as, e.g., cooperation between large numbers of unrelated subjects [7]. While several possible mechanisms have been suggested [8] that lead to assortment, i.e., to cooperative individuals being more likely to cooperate with each other, and hence to cooperation [9], they do not provide general answers and their applicability is only partial. On the other hand, the increasing body of knowledge on human interaction arising from experimental economics [10], [11] has identified many features in the observed behavior that require further explanation from a theoretical viewpoint.

One paradigmatic setup to probe different aspects of human cooperation is the Ultimatum game, invented by Werner Güth [12]. A lot of experimental research has been carried out about this game, to the extent that a moratory on further experiments was eventually suggested [11]. In fact, it is probably the only game whose dependence on anthropological factors has been addressed in depth, having been studied all around the world [13]. In the Ultimatum game, two subjects are given an amount of money. One of them, hereafter called “proposer”, chooses a division of the amount. If the second subject, hereafter called “responder” agrees, the money is divided among the two players as the proposer offered; otherwise, neither player receives anything. The game is one-shot and anonymous, so reputation is not a concern in this setup. According to economic theory, rational utility maximizers will accept any offer as responders, as a positive amount of money is better than nothing. Knowing this, proposers should make the smallest possible offer. This is very far from what is observed [11], [13]: In experiments, people rejects low offers, and proposers anticipate this and consequently propose a nearly equal division of the amount at stake. Interestingly, this behavior seems not to be exclusive of humans, and similar observations have been reported in experiments with primates [6].

While a number of cultural [1], [13]–[16] and biological influences [17], [18] have been invoked to explain the success and persistence of behavior that violates the rationality axiom, other putative explanations have been advanced on a more evolutionary tone. Thus, in a rapid evolution scenario, i.e., when reproduction takes place at a faster scale than interaction among individuals, altruistic behavior may be evolutionarily selected [19]. Large offers in the Ultimatum game arise also when subjects exhibit empathy [20], [21], a behavior that is characterized by players choosing as their offer the smallest amount they are willing to accept as responders. Another mechanism that has been studied is the existence of a spatial structure that could support the appearance of altruistic behavior [22]. Thus, it was shown that large offer levels, around a 34% of the amount to share, emerged and were stable when the population structure was a one-dimensional ring or a two-dimensional square lattice, in contrast to the convergence to the rational solution on a well mixed population. A similar result was later shown for a generalization of the Ultimatum game to describe collaborations [23]. More recently, the effect of complex (non-spatial) networks of interactions was addressed [24]–[26], finding again that large offers appeared in the population.

In line with the research we have summarized, the starting point of this paper is recent work of some of us [27] in which the spatial Ultimatum game was studied in detail. There, it was shown that under a variety of dynamics and with or without the influence of noise, quasi-empathic strategies arose spontaneously by the action of selection. This is a very important point, as in previous works [20], [21], empathy was exogenously included in the model by imposing that players would use the same amount as their offer and also as their threshold for accepting offers. We here set out to investigate in depth the connections between empathic strategies and the arising of non-rational or non-selfish behavior. As we will see below, our research program allows us to establish the two (quite general and easy to fulfill) conditions needed for the selection of empathic or quasi-empathic subjects, namely locality of interactions, i.e., that players do not interact with the whole of the population in one round (in other words, the population is not well mixed) and variance of roles, given by a possibly random exchange of the roles of proposers and responders for every player. We will demonstrate below how these two conditions lead to the arising and dominance of empathy in a time lapse that depends on the existence and degree of highly connected players and which, for socially applicable situations, is not large.

## Model

Our model has interaction locality among its main ingredients, albeit, as we will see, we will not need context preservation as in [28], i.e., players may interact with different partners at every round in so far as they do it with a small subset of the whole population each time. In any event, to make clear the presentation of our model and results, we begin by considering a set of agents playing the Ultimatum game on a network, i.e., they are located on the nodes of networks and play the game only with their neighbors. Without loss of generality, the total amount to be shared will be hereafter normalized to the unity, so we can characterize the strategy of every player by two parameters, 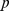, 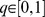. The value of 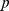 indicates the fraction of the reward offered by the player when acting as proposer, whereas 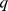 represents the acceptance threshold, i.e. the minimum quantity that the player accepts when acting as responder. It is important to note that we will let both parameters evolve independently, in other words, we will never impose exogenously that players are empathic, which would correspond to offering the amount one is ready to accept, i.e., 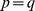.

With this notation, an interaction between players 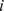 and 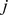, with 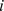 taking the role of proposer and 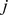 that of the responder is given by (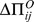 and 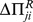 stand for the increment of payoff for 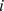 and 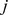, respectively):
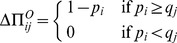
(1)

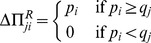
(2)


Every time step, we go through the whole network sequentially, and every player participates in an Ultimatum game with each of her neighbors. Roles (proposer and responder) are assigned to the players randomly in every encounter, so that the same individual can act twice as proposer or respondent with the same opponent within the same time step of the simulation. For comparison when needed, we also consider a non-random assignment rule, in which the focal agent plays as proposer (and therefore she plays as responder when her neighbors are the focal agent). We always use the random assignment procedure unless otherwise stated. In this case, the expectation for the payoff increment of 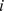 after a double interaction with 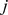 is
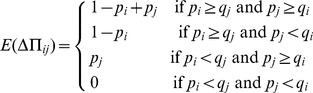
(3)In case non-random roles are assigned, the expectation value given by Eq. (3) becomes the exact payoff change after a double 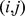 interaction. The final payoff is the sum of the increments after playing with all neighbors.

After every agent in the network has played, they update their strategies. Each individual compares her final payoff with that of her neighbors and modifies her parameters 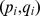 according to a well-known procedure called *proportional imitation*: Player 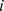, the one whose strategy is to be updated, selects one neighbor 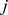 at random. Then, provided that the selected neighbor's payoff is greater than that of player 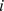, her strategy, i.e., her values of 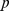 and 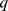, will be adopted with probability
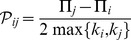
(4)where 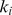 and 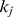 are the number of neighbors of 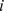 and 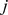 respectively. The denominator serves as a normalization factor in order to ensure that 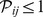. This update rule is also known as (discrete) “*replicator rule*”, since in the limit in which the number of agents and their degree go to infinity it leads to the famous replicator equation [29] for the continuum time evolution of the distribution density of strategists, as shown by [30], [31]. To mimic the effects of mutation (otherwise the dynamics is purely imitative and not innovative, so new strategies cannot be introduced in the system), a small amount of noise has been implemented by adding to the copied parameters a random value uniformly distributed in 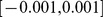 (not necessarily the same for 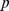 and 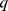). We note that this in fact represents small mutations, whereas in other evolutionary game models larger mutation rates have been explored [32]; as in the latter case nontrivial phenomena arise due to the mutation process and not to the game itself, we have chosen to focus on the small mutation limit in order to elicit effects originating purely from the Ultimatum game.

As one of our main aims is to study the effect of having different neighborhood sizes, we need to monitor the effects of heterogeneity in the degree distribution of the network in a controlled manner. To this end, we resorted to introducing networks with a log-normal degree distribution, which has the advantage of having a bounded variance that can be controlled independently of the mean (note that the Poisson distribution has a variance that depends on the mean, this being the reason why we have not used this distribution). Unless otherwise noted, simulations were done on 20 networks and 1000 realizations of the process per network, for 10^5^ generations for each parameter set. Networks had 2500 nodes. We considered the following choices for the heterogeneous networks (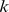 denotes the degree):

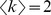
, 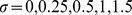.

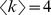
, 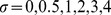.

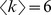
, 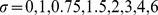.

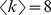
, 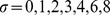.

Note that, for each value of 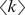, the variance is chosen in such a way that, when a list of nodes and degrees is generated from the distribution to feed in a configurational algorithm [33], a giant component comprising the majority of the nodes can emerge.

Finally, as we mentioned above, the ingredient of locality does not require that the opponents are always the same. To check this point, we have also studied the case in which the interaction network is not fixed but instead players exchange randomly their locations. In those simulations, after every round of the game and the subsequent strategy update, all players are reshuffled with the constraint that their degree is kept constant, so any new effects that might arise do not come from a change of the number of neighbors but rather from the fact that the neighbors themselves changed.

## Results

### Emergence of empathy

**Figure 1:**
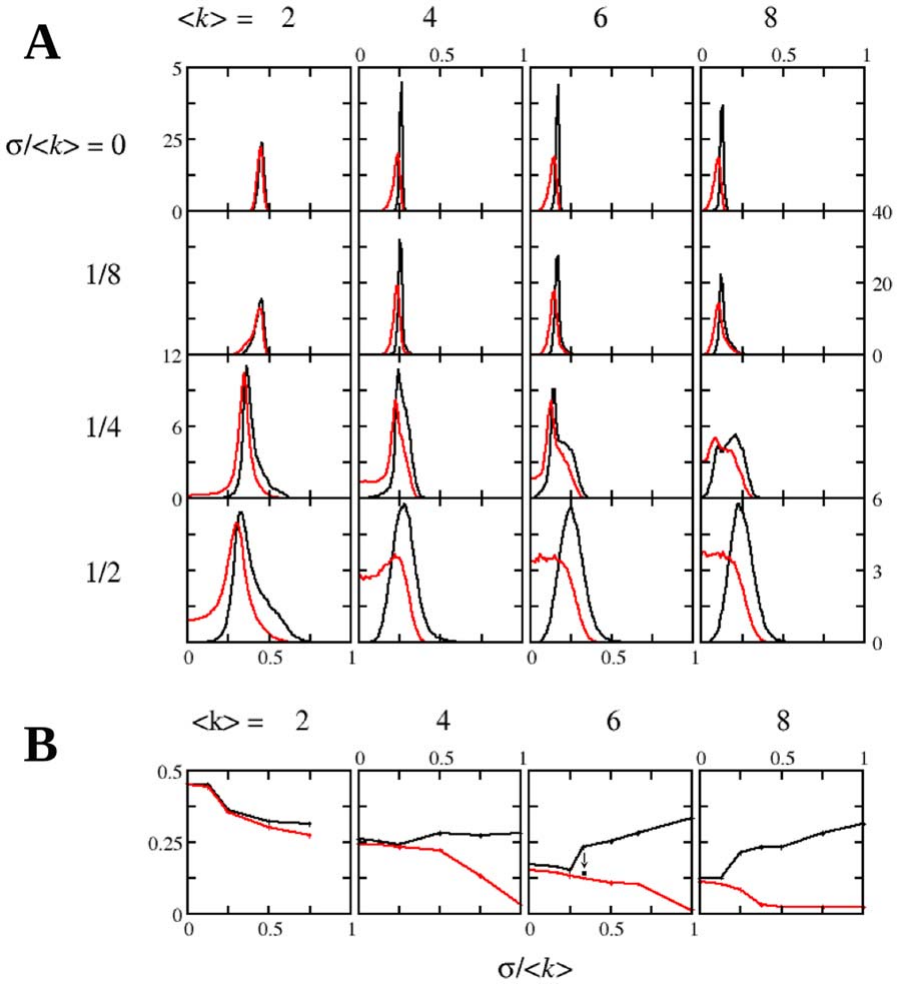
Dependence of the quasi-empathic behavior on the network characteristics. A. Distributions of the strategies present in the population of Ultimatum game players after long simulation times. Black line, distribution of offers; red line, distribution of acceptance thresholds. Players evolve on networks with log-normal degree distributions, mean and variance being as indicated in the plots. System size is 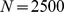, and averages are taken over 20 different realizations of the game on 1000 networks up to 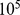 games per player. B. Mode of the distributions in A as a function of the network heterogeneity. The black square in the plot for 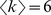 indicates the observed trend of the data as simulation times grow (obtained after 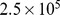 games per player).

We begin reporting on our results by introducing the one-dimensional distribution density of offer, 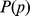, and acceptance thresholds, 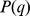, on networks with different mean degree and degree variance, summarized in Fig. 1A. In these plots we present both distribution densities after a long time has elapsed (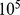 games per player) and there are no apparent further changes to the distributions. The main result that is apparent from the figure is that in all cases the distribution of offers and of acceptance thresholds are very close to each other, acceptances always a little bit to the left of offers. Having smaller acceptance thresholds than offers makes sense because in the opposite case many instances of the game would lead to no gain for the players. In addition, we checked that the result is true not only in terms of the distributions but also for every individual player: In the final state, the population consists of empathic players, with very similar values for both parameters (and verifying the same property, acceptance being smaller than offer). This remark notwithstanding, the plots show a clear difference between the case with 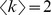 and higher values for the mean degree. The former exhibits a clear peak in the regular case (zero variance of the degree, implying all nodes have degree 2) that widens and moves to the left for higher variances. Higher values of the mean allow to explore more inhomogeneous networks, and the results seem to show new phenomenology: Beginning with the regular case, which is similar to the 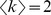 network (cf. Fig. 5d in [27] for the 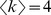 system), the peak first evolves, when the value of the variance increases, as in the previous case, but later it returns slightly to higher values of the parameters. Subsequently, the acceptance threshold distribution ends up developing a shoulder in the region of small values of the parameter. This means that the population becomes more heterogeneous, with more rational individuals appearing (very low acceptance thresholds) but it should be remarked that many individuals still remain quasi-empathic as in the 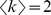 networks. Both observations are in agreement with the experimental observations [13].

In order to assess in more detail the differences between homogeneous and heterogeneous networks, Fig. 1B shows the dependence of the offers and the acceptance thresholds distribution with the network heterogeneity by looking at the mode of the distribution. We observe that networks with very little or no heterogeneity evolve to quasi-empathic behavior, with 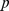 and 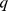 very similar to each other (but always 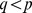). However, networks with larger degree variances show a different situation: Due to the appearance of the shoulder mentioned above in the acceptance threshold, its mode decreases steadily and seems to approach zero with increasing heterogeneity. The non-monotonic evolution of the distribution of the offers can also be clearly appreciated, first decreasing and then increasing, so that the mode of the offers separates apart from that of the acceptances, with increasing heterogeneity. Thus, one would conclude from Fig. 1B that empathy arises only in homogeneous or (at most) slightly heterogeneous networks.

The above conclusion is, however, proven wrong when looking into more detail at the dynamics of the evolutionary process. Indeed, the experience of previous work [26] on this game over heterogeneous networks suggests that, as far as degree heterogeneity allows for the presence of hubs, the convergence to the asymptotic state of the evolution slows down dramatically, for they have a large probability of keeping whatever strategy they had initially, due to the large payoffs they can accumulate. It is important to clarify that the simulations in that previous work did not include the randomization of the proposer/responder roles. Therefore, for the purpose of this work, we have checked with new simulations that including it does not change the results reported there, as indeed occurred. Thus, we can safely claim that, as in [26], hubs still control the evolutionary dynamics also when random assignment of roles is implemented. In our case, though the log-normal degree distribution have only realizations with”moderate” local hubs, in the extent that these nodes are degree-dominant, which easily translates into higher resilience to invasion, one might be able to, by looking at longer (albeit more expensive in computational terms) simulations, check for the time scales characteristic of local hubs invasion.

In agreement with these expectations, our results for simulation times up to 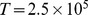 updates per player (for a network with 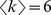 and several degree variances), show clearly that the evolution of the mode of 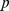 is slower than that of the mode of 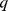 in all cases. This suggests that for the largest values of the variance, the averaged results for the mode of the offers shown in Fig. 1B, showing its increase versus heterogeneity, are likely to be an artifact due to the larger time scale of local hubs invasion processes. As a check, we re-run simulations for a specific set of parameters, finding the black square shown in Fig. 1B, finding that indeed the mode of the offers approaches that of the acceptance thresholds for longer runs.

**Figure 2:**
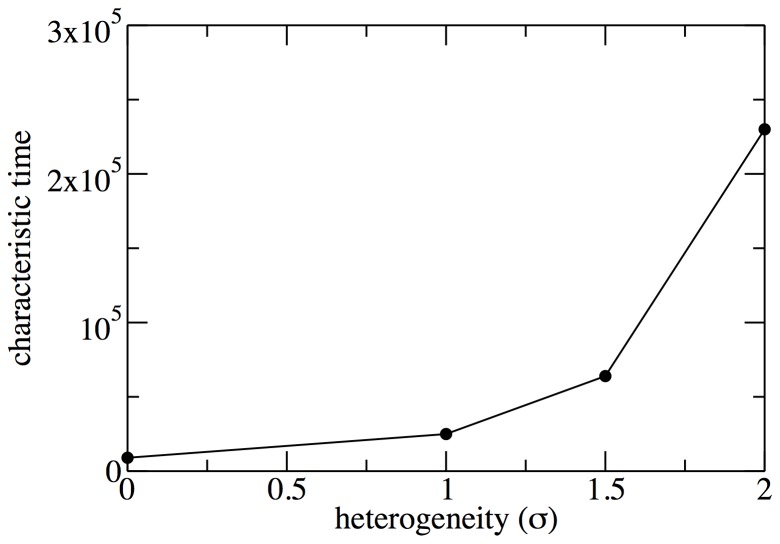
Characteristic convergence time to quasi-empathy. The plot presents data from simulations on networks with 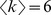 as a function of the degree variance.

As further support for our discussion, Fig. 2 shows the characteristic time for the establishment of empathy as a function of the degree of heterogeneity. We have checked that the dependence on Fig. 2 is super-exponential, i.e., it grows very fast with the degree of heterogeneity. This is an interesting feature on which we will comment more below. What becomes apparent from this analysis is that if the networks were given enough time to converge, quasi-empathy would always be the asymptotic result for any degree of heterogeneity (within the limited values of heterogeneities that we are considering). It is important to note that situations like the ones previously studied, in which convergence to steadiness has not been fully achieved, are however of the utmost interest, since in general, networks also evolve and therefore their “static” topology can not be considered so forever.

### The need for local interactions

In order to get more insights into the mechanisms behind our observations on the emergence of empathy, we believe it is important to check which part of the quasi-empathic behavior arises from the network structure and which one comes only because interactions are local. Here local means precisely that the number of interactions per player and unit time is small, very far from the system size, and not “short range” as it usually means.)

To that end, we studied the case in which after every time step all players are randomized, keeping the network constant. We considered again 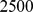 nodes on a regular (i.e., null degree variance), random network of degree 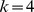. The reshuffling procedure means that after a moderate number of interactions, a player has interacted with a large part of the population, thus mimicking somewhat the behavior of a well-mixed population. As a result, in this scenario the network structure is lost while players keep interacting with a limited number of partners at each round. This means that the neighboring set of a player is, for each time step, randomly sampled from the whole network, which is a fast diffusion condition. The difference with a well-mixed case is only that the agent plays with a small sample (far from 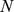) from the whole population, so that fluctuations in the sampling can be large.

**Figure 3:**
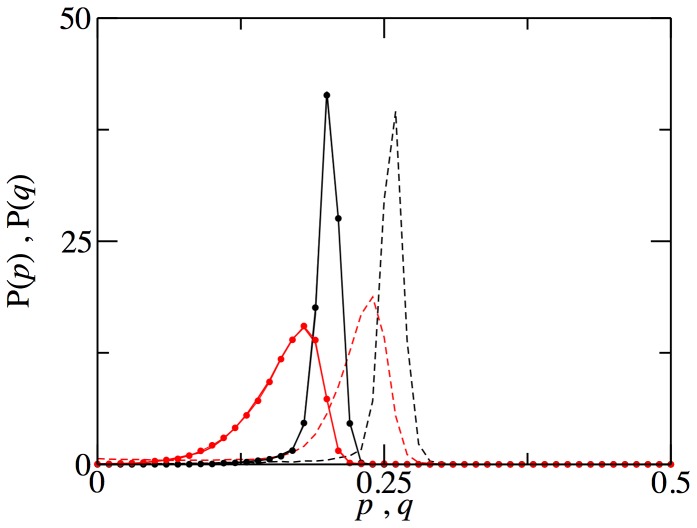
Probability distribution of the strategies present in the population in the steady state. Ultimatum game players evolve in random networks, keeping the neighbors fixed (dashed line) or reshuffling them after every instance of the game (solid line). Black line, distribution of offers; red line, distribution of acceptance thresholds. Circles show the results in a ring network with reshuffling (notice that they overlap completely with the solid lines). Mean degree of the networks is 4, and other parameters are as in Fig. 1.

As can be seen from Fig. 3, the fact that neighbors are different in different time steps does not change the results qualitatively; there is only a displacement of the distributions of offers and acceptances to the left, but still they keep very far from the rational behavior. Note that as the network has a zero degree variance, there is a unique invasion time scale involved, so that these results are cleanly free from the presence of larger invasion time scales that plague the evolutionary dynamics of heterogenous networks.

Fig. 3 also shows the comparison with a 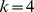 ring with player reshuffling, which leads to exactly the same results. Thus, we can safely conclude that it is the fact that interactions are restricted what leads (or suffices) to quasi-empathy, and that context preservation, i.e., having always the same neighbors [28] would only have the effect of increasing the asymptotic values of the two parameters, so driving the quasi-empathic evolutionary outcome a bit farther from rational behavior (which is also empathic, after all, from empathy definition).

We have also analyzed possible influences from other factors, such as clustering. For that purpose, we studied a ring network in which every node is linked to its second and third neighbors, thus having zero clustering by construction, obtaining again the same results as for the usual ring (not shown). In fact, even when keeping the neighborhood fixed for the whole simulation, the results on both rings are the same, which leads us to question the conclusions of other works such as [24] which suggested that the existence of triangles was the reason for the high levels of acceptance thresholds and offers observed in some networks.

### Dynamics of empathy establishment

**Figure 4:**
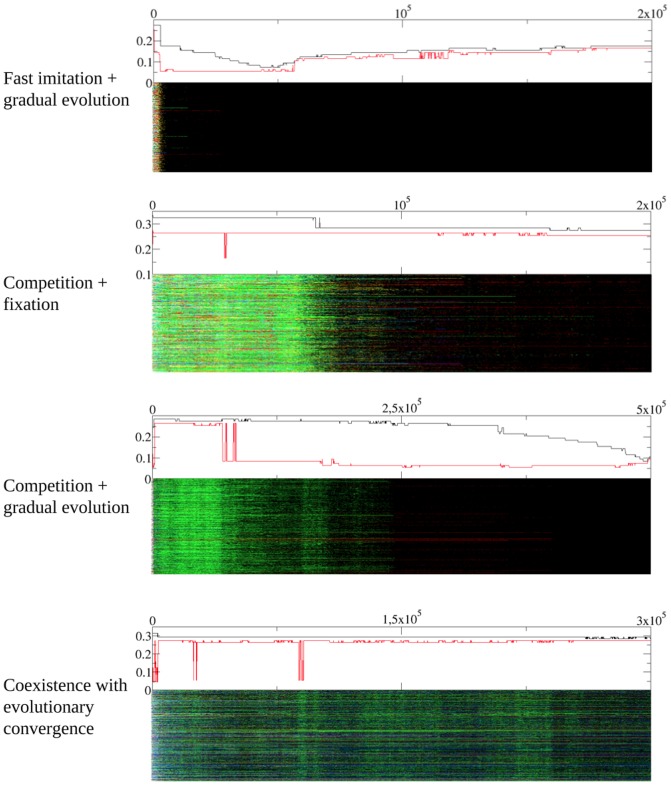
Time evolution of the parameters 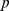 and 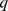. From top to bottom: 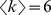, 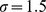 (first two plots); 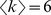, 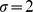 (third and fourth plots). Every point in the vertical axis represents a player and evolution takes place along the horizontal axis. Every strategy (identified by 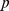 and 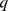 is assigned a random color) and followed until it is replaced by other one (hence adopting its color) or until the end of the simulation. For every plot, the upper graph represents the modes of 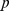 and 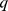 and the lower graph represents the individual strategies.

We have explored the dynamics of heterogeneous networks in more detail. Examples of the time evolution of specific realizations are shown in Fig. 4. In these simulations, we have followed the population genealogy in the following way: we assign a label to every individual at every generation indicating from whom has she taken her current strategy. This is subsequently represented by colors. In the figures we represent the modes of 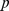 and 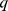 in the upper plot and the whole genealogy in the lower plot. As is immediately apparent from the plots, one can have very different situations along the time evolution of the strategies. To begin with, Fig. 4 shows a case in which the whole population imitates very rapidly a common ancestor (black) whose strategy is not empathic. This is then followed by a convergence of 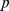 towards a quasi-empathic behavior. In a second scenario, the second graph depicts the competition between two strategies (black and green) that leads to the fixation of the most quasi-empathic one and a freezing of the evolution. The next graph, obtained on a slightly more homogeneous network (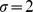 instead of 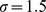 in the rest of the plots) presents a realization with a long competition followed by fixation and convergence towards quasi-empathy, whereas finally in the last plot we observe evolutionary convergence towards a set of strategies that are very similar and, once again, quasi-empathic.

**Figure 5:**
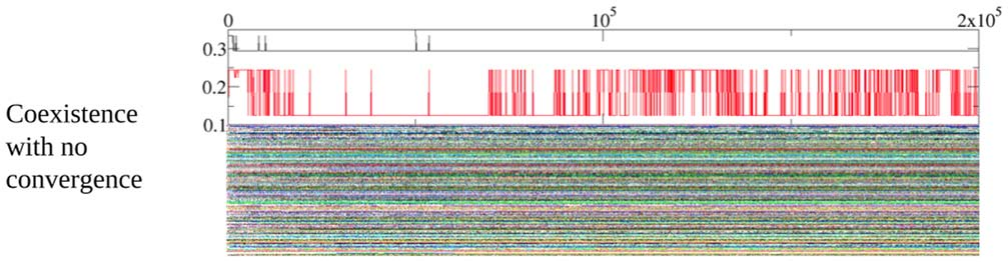
Time evolution of the parameters 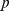 and 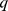. Every point in the vertical axis represents a player and evolution takes place along the horizontal axis. Every strategy (identified by 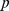 and 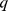) is assigned a random color and followed until it is replaced by other one (hence adopting its color) or until the end of the simulation. For every plot, the upper graph represents the modes of 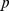 and 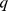 and the lower graph represents the individual strategies. In this specific realization, there was no random assignment of roles in the game, so every player played once as a responder and once as a proposer with all her neighbors.

All the examples shown in Fig. 4 share a common feature, namely that evolution takes place in a punctuated manner. We believe that this kind of evolution is very likely to arise from the hubs of the log-normal network, that are less-prominent than in a scale-free network, but still they are hubs. It is important to point out in this respect that here the need for randomization of the proposer/responder role is crucial; without it, one can calculate (see below) conditions for a hub to be invaded that turn out to be very restrictive, rendering it impossible in practice to invade it (see below). Therefore, without this randomization the networks would be eventually frozen prior to convergence to quasi-empathy. In order to make this point clear, Fig. 5 depicts a simulation without random assignment of roles. It can be easily realized that in this case there is no convergence, and very many strategies remain alive in the network. In this respect, it is important to note that coexistence of strategies also appears in the random case, but the system is not frozen and evolutionary convergence is still at work in the system (cf. Fig. 4d). In the case with no randomized roles, the evolution is completely stopped and no evolutionary convergence is observed.

To shed further light on the need for randomness for the convergence to quasi-empathic strategies to occur, we have carried out a simple analytical calculation that allows to understand the dynamics of the process and the freezing of the evolution. We consider here a double star network, consisting of two central nodes or hubs, 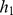 and 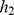, connected to each other. On their side, 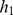 is connected to 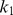 peripheral nodes and 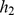 to 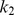 nodes, with 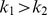, so we will call the former the main hub and the latter the secondary hub. We are interested in the invasion process of a node by a strategy with a smaller offer, in order to assess the possibility of convergence towards a quasi-empathic strategy; therefore, we will assume that 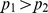, albeit the results are valid in general without this constraint. We will denote 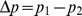. As for the 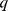 values, we will only assume that they are smaller than their respective 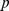 values, and hence all instances of the game succeed and the payoffs are shared as proposed by the proposer. In what follows we will consider two different situations: the generic one, in which nodes linked to 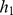 share its strategy, and the extreme one, in which all nodes but 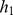 have the strategy of 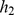.

We will begin by analyzing the deterministic (non-randomized roles) case. In the generic case, the payoff difference between the secondary and the main hub is 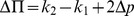. With this result, it follows that the main hub will not be invaded if 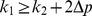, and as the maximum reasonable value for 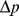 is 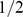, it is clear that the main hub will keep its strategy. The situation most favorable for invasion is the extreme one, in which we find that 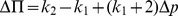. The resilience condition for the main hub is now 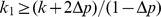. Assuming again that 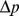 can be at most 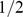 in a normal case, we have that the hub cannot be invaded if 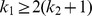. Such a strong condition is verified in instances of scale-free networks (although due to the rich-club phenomenon, in a preferential attachment network hubs are closer in degree); however, in our setup of log-normal networks, this condition is difficult to fulfill and therefore hubs can be invaded in such an extreme situation. In any event, it becomes apparent that invasion requires differences 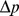 quite large, and that invasion by a very similar strategy may be exceedingly difficult. In particular, the small differences produced by errors (mutations) during the update of strategies are unable to spread and therefore gradual evolution cannot take place. This results in a punctuated evolution driven by imitation of the initial strategies.

When there is random assignment of roles in every game, the maximum payoff difference between the two hubs takes place when the secondary is always the proposer and the main hub is always the responder. This modifies the payoff difference, that now becomes 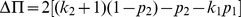, and the non-invadability condition is 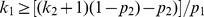. This condition is valid in the generic case, but if the neighbors of the main hub have already been invaded by the strategy of the secondary one, the condition becomes 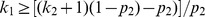. This implies that for relatively small values of 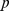 invasion is possible in networks that are not too heterogeneous (for instance, if 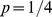 we need 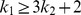 for non-invadibility, which is a very stringent constraint). Gradual evolution is also possible, as the non-invadability condition does not depend on 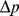 now (although the requirement that the role is the same in the two interactions for both players makes it somewhat slower). This is in agreement with the need for long time of evolution in order for the system to reach an asymptotic state, and with the characteristic time increasing with the heterogeneity of the network.

We can now suggest what is the situation in a general network. Usually, a network has several main hubs that, if not connected to each other, can only be invaded through secondary hubs. When evolution is deterministic, convergence through imitation can only be achieved up to a certain point in 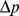, specifically until 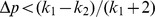; smaller differences do not allow to invade a main hub in view of the above discussion. On the contrary, when there is a random assignment of roles, evolution is possible in so far as there are nodes with 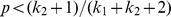, and gradual dynamics can take place if the initial condition contains a 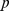 that verifies this condition. All in all, without being a rigorous proof, this calculation shows that in a deterministic setting convergence is very difficult if not plainly impossible, whereas in the random dynamics even large hubs may be amenable to invasion, possibly in a long but still attainable time. This fully agrees with the picture our simulations above are providing.

## Discussion

In this paper, we have studied the spontaneous emergence of empathy in a population of players playing an Ultimatum game with a subset of the population, be it fixed on a network or simply randomly chosen at every round. So far, empathy is something that has been imposed a priori in many previous works. The results from the works that restrict consideration to empathic strategies, i.e., 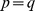 [give refs.] made it clear that empathic strategists with significantly positive offers and acceptance thresholds (say fair strategists) resist well the competition with rational behavior (empathic but unfair), under some conditions: *Empathy promotes fairness*, as simply said. Note that rational behavior is dynamically accessible inside the set of empathic strategies. (Also is the most irrational behavior 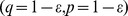, albeit evolution is never driven there, being the observed evolutionary outcomes almost always below the perfect fairness 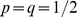). The observed emergence of fairness from empathic conditions could ultimately be rationalized from the observation that two players using the same arbitrary empathic strategy in a double interaction with interchanged roles do as good as the best possible arbitrary empathic combination, for both deals (proposer1, respondant2) and (respondant1, proposer2) are materialized, independently of the particular 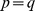 value. However, as non-empathic (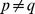) strategy perturbations are allowed, the well-doing of an empathic dimer could be menaced by the individual advantage (or neutrality) of unilateral deviations from fair empathy.

Indeed, if one leaves aside the empathic restriction, so that general 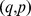 strategies are allowed to compete, for a well mixed finite (up to sizes of 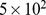 players) populations simulations [20] show the evolution of *mean* offer and acceptance threshold towards their rational values (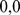). For the macroscopic well-mixed limit, Seymour [34] derived a replicator partial differential equation (PDE) by invoking a setting in which a population of (only) proposers offer their deals to a population of (only) responders. In this setting, the distribution densities 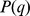 and 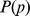 satisfy a separable two-variable continuum replicator PDE with families of stationary solutions in which the offers' density 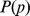 is a point density concentrated at an arbitrary offer 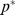, and 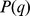 is arbitrary with support inside 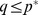. Arbitrarily small levels of white noise diffusion seems to suffice, however, to make these densities drift down to the rational solution, while other different types of noise (*e.g.*, asymmetric regarding proposer-responder roles) could lead to other non-rational stationary (or at least very slowly evolving) densities.

In contrast to those previous works, in the present one we are concerned with networked populations of ultimatum game strategists that replicate. We make here the observation that the evolutionary dynamics of the ultimatum game in networks leads spontaneously to relatively fair quasi-empathic macroscopic states (*i.e.*, asymptotic densities 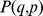 of the continuum strategies). The numerical investigation on the minimal conditions that seem to suffice to attain a long time quasi-empathic behavior (relatively) far from rationality shows that two features are of capital importance. To begin with, the restriction to proposer-responder symmetry of the double interaction at each time step may impede the convergence to quasi-empathy due to freezing of invasion of hubs, that are easily surrounded by imitating strategists of its random initial (possibly non-empathic) strategy. We argue that, though *in average* every player acts as proposer as many times as she plays as responder, the simple randomization in the role of proposer/responder of each players' pair encounter, is effective in terms of facilitating the scape from fixation of non empathic strategies in the neighborhood of (even moderately) highly connected nodes, i.e., it makes (moderate) hubs “invadable”. The randomization of roles that we have used does not violate the individual's symmetry of roles “on average”, though it allows for random walks around strict instantaneous symmetry. On the other hand, we also found the all-important ingredient of restricted interaction. Thus, interaction in small (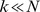) neighborhoods (even when they change every time step, in the sense of network rewiring each time step, and zero degree variance network) also favors the evolution towards fair empathic distribution densities, with fairness decreasing when the average number 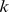 of interactions per player and time step grows. This fact fits well with previous simulations for well-mixed finite populations.

This finding, namely that interactions in small groups involving some degree of randomness in the assignment roles is enough for empathy to emerge, is a most relevant contribution to the literature as it opens the door to understand experimental results on the Ultimatum game. As we have shown, evolution under such a widely applicable setting leads to empathy, and empathy, in turn, leads to fairness. It is important to stress that, even if the present results have been obtained only for the discrete analog of the replicator dynamics, we believe that, in view of previous works [26], [27], the same behavior will be observed for other imitative dynamics, be they imitate-the-best, social imitation, or any other involving copying the behavior of a partner (possibly with mutation). Other update rules not based on imitation, such as best response or reinforcement learning, might lead to different behaviors in so far as they are based in the player's own cognitive capabilities. This is an interesting question that deserves further research. Finally, another question that arises naturally concerns the symmetry of proposer-responder roles. Lack of symmetry of roles is a practical situation in some economic or social settings, for there are people more akin to propose a deal to others, as well as others which act more frequently as responders. We have seen that the fluctuations in the interchange of roles help to overcome the dynamical trapping on “metastable” states of evolution. Then the lack of interchange symmetry may, at least dynamically, have an influence on the evolutionary dynamics, at some time scales, not only on the Ultimatum game, but in more general settings. Research into this issue would be greatly needed to ascertain the extent as to which symmetry considerations are relevant.
